# SpheriCal^®^‐ESI: A dendrimer‐based nine‐point calibration solution ranging from *m*/*z* 273 to 1716 for electrospray ionization mass spectrometry peptide analysis

**DOI:** 10.1002/rcm.9035

**Published:** 2021-01-21

**Authors:** Joakim Romson, Oliver Freiholtz, Anher Saeed, Adrián Soto Kronberg, Athea Thomas, Jamie Godfrey, Michael Malkoch, Scott M. Grayson, Åsa Emmer

**Affiliations:** ^1^ School of Engineering Sciences in Chemistry, Biotechnology and Health, Department of Chemistry, Division of Applied Physical Chemistry, Analytical Chemistry KTH Royal Institute of Technology Stockholm Sweden; ^2^ School of Engineering Sciences in Chemistry, Biotechnology and Health, Department of Fibre and Polymer Technology, Division of Coating Technology KTH Royal Institute of Technology Stockholm Sweden; ^3^ Polymer Factory Sweden AB Stockholm 11428 Sweden; ^4^ Department of Chemistry Tulane University New Orleans LA 70118 USA

## Abstract

**Rationale:**

A calibration solution for mass spectrometry needs to cover the range of interest with intense and sufficiently narrowly spaced peaks. Limited options fulfilling this may lead to compromises between performance and ease of use. SpheriCal^®^‐ESI was designed to combine high calibration performance for electrospray ionization (ESI) mass spectrometric analysis of peptides in positive mode with quick and easy use.

**Methods:**

The developed calibration solution was tested using three mass spectrometers: two ion traps and one tandem quadrupole. The *m*/*z* errors of SpheriCal^®^‐ESI itself and of a tryptic digest of cytochrome C were measured after calibration. The results were compared with those achieved with ESI Tuning Mix. The memory effects of the dendrimers, and contamination from Na^+^ in the calibration solution, were evaluated.

**Results:**

SpheriCal^®^‐ESI showed good shelf life as powder and was quickly reconstituted for use. Achieving intense and stable signals was straightforward. The accuracies and precisions were as expected for the instruments. SpheriCal^®^‐ESI was more precise and at least as accurate as ESI Tuning Mix. The memory effects and Na^+^ contamination were found to be negligible in typical peptide solvents. In addition, the dendrimers showed predictable dissociations with product ions common to collision‐induced dissociation in both ion trap and tandem quadrupole mass spectrometers.

**Conclusions:**

SpheriCal^®^‐ESI provided easily accessible calibration by showing intense signals at low infusion rates and at source settings equal or similar to those used in peptide analysis. Nine calibration points in the range of interest gave precise and accurate results. Memory effects and contamination were negligible even without rinsing.

## 1 INTRODUCTION

Good calibration of the *m*/*z* axis is of utmost importance for accurate *m*/*z* measurements of analytes. A multitude of calibration solutions for different requirements are available today. A suitable calibration solution should cover the range of interest, with intense and stable peaks with sufficiently narrow spacing between them. From a user perspective, it is advantageous if preparing the solution and obtaining the signal require little effort, and if memory effects and source contamination are minimal.

Manual calibration may be preferred as the operator can distinguish the monoisotopic peak, notice a poor or unstable signal, or a distorted peak shape, and discriminate against noise. Automated calibration, on the other hand, should be more accurate and precise in establishing peak apex or centroid. Automated calibration should always be checked to ensure that the correct (monoisotopic) peak is chosen and not distorted.

Although an isotope pattern provides a means of confirming molecular charge and distinguishing a molecular signal from noise or artifacts, some existing calibrants such as clusters of CsI^1^ are monoisotopic, avoiding the possibility of erroneously picking an isotope peak. CsI does not necessarily cause memory effects,^2^ but the issue of source contamination has been raised.^3^ Other salt clusters have been suggested,^3^, ^4^ as well as water clusters.^5^ However, clusters seem to depend on source parameters significantly different from those used in, for example, peptide analysis, at least at higher *m*/*z*.

Poly(ethylene glycol) (PEG) and similar polymers provide closely spaced peaks, but show persistent memory effects.^3^

Ultramark^6^ 1621 shows evenly spaced intense peaks with the monoisotopic peak being the most intense also at high *m*/*z* due to fluorination, but it covers only *m*/*z* 900–2200 and can show memory effects.^4^ In addition, concerns of potential biological hazards from fluorinated organic compounds have been raised.^7^ Nevertheless, these and similar molecules are common in calibration mixtures, such as ESI Tuning Mix and Pierce™ LTQ ESI Positive Ion Calibration Solution.

If peptides (typically stored in a freezer and often having a limited shelf life^8^) are used for calibration, potential memory effects for subsequent peptide analysis may result in false positives, even in MS^*n*^ spectra. In addition, as was found here in the case of a protein digest, peptides may show ambiguous signals.

Dendrimers are already used for calibration of matrix‐assisted laser desorption ionization mass spectrometry (MS) instruments,^9^, ^10^ and have been suggested as internal calibrants in electrospray ionization (ESI).^11^ The ester‐based dendrimers used here tend to ionize as metal–ion adducts rather than protonated species, as opposed to nitrogen‐containing molecules, such as peptides, that tend to be protonated. The nature of dendrimers allows tailoring the properties by choosing different end groups, and modulating the size by the number of generations used. By using a rather compact ester core and relatively hydrophobic end groups, multiple charges may be disfavored due to charge repulsion. Here, a dendrimer‐based calibration solution for external calibration for peptide analysis is presented: SpheriCal^®^‐ESI. This is demonstrated to yield satisfactory signals for accurate and precise calibration, and also for demanding applications. Its convenient preparation, good ionization using the same or similar source settings as in peptide analysis, and negligible memory effects ensure low operator effort.

## 2 EXPERIMENTAL

### 2.1 Chemicals

SpheriCal^®^‐ESI dendrimers (structures are available in the supporting information) were provided by Polymer Factory Sweden AB (Stockholm, Sweden). MeOH, MeCN, and NaHCOO were purchased from Merck (Stockholm, Sweden). HCOOH was purchased from Fluka (Stockholm, Sweden). NH_4_HCO_3_ was purchased from AppliChem (Stockholm, Sweden). Water was purified in a Synergy 185 system (Millipore, Bedford, MA, USA) to a resistivity of 18.2 MΩ cm at 25°C. ESI Tuning Mix, PEG‐600, tetrahydrofuran (THF), cytochrome C (CytC; *Equus caballus*), and trypsin (porcine pancreas, TPCK‐treated) were purchased from Sigma Aldrich (Stockholm, Sweden).

### 2.2 Sample preparation

Stock solutions of the dendrimers in THF and of NaHCOO in H_2_O were added to Eppendorf tubes (Hamburg, Germany) and the solvent was removed using an Eppendorf concentrator 5301. The tubes were stored on the benchtop until use, when MeOH was added, yielding the calibration solution (SpheriCal^®^‐ESI; Table 1). Tryptic digestion of CytC was performed at 1.0 mg/mL CytC with 5 wt% trypsin in 10 mM NH_4_HCO_3_ at 37°C for 18 h, then terminated at 95°C for 5 min. The digest was analyzed in MeCN (60 vol%) with HCOOH (0.1 vol%). ESI Tuning Mix was diluted 20 times into 5 vol% water in MeCN. (Previously, poor intensities of the lower *m*/*z* signals of ESI Tuning Mix have been observed. Therefore, five times higher concentration than default was used here to ensure intense signals.) PEG‐600 was dissolved in MeOH to 10 and 50 μg/mL.^12^

**TABLE 1:** Composition of the SpheriCal^®^‐ESI calibration solution, and calculated monoisotopic *m*/*z* values

Component	Concentration (μg/mL)	Ion	Calculated *m*/*z*
NaHCOO	5.0	—	—
S273	1.0	[C_14_H_18_O_4_ + Na]^+^	273.109730
S493	0.5	[C_26_H_30_O_8_ + Na]^+^	493.183289
S632	20	[C_39_H_76_O_4_ + Na]^+^	631.563582
S755	0.5	[C_41_H_48_O_12_ + Na]^+^	755.303798
S888	40	[C_53_H_100_O_8_ + Na]^+^	887.731041
S1086	5.0	[C_65_H_122_O_10_ + Na]^+^	1085.893021
S1195	0.5	[C_65_H_72_O_20_ + Na]^+^	1195.450915
S1325	40	[C_40_H_27_O_10_I_5_ + Na]^+^	1324.672003
S1716	5.0	[C_92_H_108_O_30_ + Na]^+^	1715.681763

### 2.3 Instrumentation

Development of the SpheriCal^®^‐ESI calibration solution was performed in “Maximum resolution” mode (500 *m*/*z* units/s) using an obsolete Bruker Esquire‐LC ion trap mass spectrometer with esquireControl 6.16 (Bremen, Germany). Using the obsolete instrument ensured that the calibration solution would give intense peaks even for unfavorable applications. In addition, to further emulate unfavorable conditions, the low flow rates used have the advantage of minimizing economic and environmental impact, and source cleaning. Calibration was additionally evaluated using a modern Bruker amaZon speed ion trap with trapControl 8.0 and an older Waters Micromass Quattro micro tandem quadrupole using MassLynx 4.1 (Waters, Milford, MA, USA). For comparison, the performance of the recommended calibrant, ESI Tuning Mix, was evaluated using the amaZon. Parameters for ion guides, ion traps, quadrupoles, peak picking, and smoothing were set at or similar to default values. No extensive optimization of any parameter except the SpheriCal^®^‐ESI composition was performed. Ion trap data were analyzed using DataAnalysis 4.2 (Bruker). Quattro micro data were analyzed using MassLynx 4.1. Theoretical *m*/*z* values and isotope patterns were calculated with enviPat^13^ and ChemDraw Professional 16.0 (PerkinElmer Informatics Inc., Waltham, MA, USA). Plots and statistical evaluations were made with Excel 16.0 (Microsoft Corp., Redmond, WA. USA).

### 2.4 Calibration procedure

Automated calibration was confirmed on all instruments but manual calibration was used on the traps: 50 *m*/*z* unit ranges around the calibrant peak were scanned, averaging 100 scans per spectrum and placing the calibration in the middle of the peak at FWHM, using a real‐time Gaussian smoothing of 0.05 *m*/*z* units. Calibration with the tandem quadrupole was automated (manual calibration was not available), calibrating both quadrupoles at low (180 *m*/*z* units/s) and high (900 *m*/*z* units/s) scan speeds, and at static scan, 1 min each. The scan window was 2 *m*/*z* units and the expected error 1 *m*/*z* unit, smoothing was 2 passes at 0.75 *m*/*z* units Savitsky–Golay, and centroid peak picking at 80% area.

### 2.5 Instrumental parameters and evaluation of calibration

Evaluation of *m*/*z* accuracy and precision was performed by calibrating S273–S1716 or *m*/*z* 118, 322, 622, 922, and 1522 of ESI Tuning Mix, then scanning *m*/*z* 200–2000 and recording spectra of SpheriCal^®^‐ESI or ESI Tuning Mix immediately afterwards. Then, after a few minutes of standby for syringe change and rinsing of the liner, a tryptic digest of CytC was recorded (theoretical *m*/*z* values calculated from uniprot ID^14^ P00004 using PeptideMass,^15^ allowing two missed cleavages). This procedure was repeated five times for each instrument (ESI Tuning Mix was only evaluated with the amaZon) using parameters as follows.

On the amaZon/Esquire “Maximum resolution” mode (5100/500 *m*/*z* units/s) was used, with 50/20 scans per spectrum, apex peak picking with peak width 0.1/0.2 *m*/*z* units at FWHM, and the Quattro micro at unit resolution at 180 *m*/*z* units/s, 3 scans per spectrum, processing as during calibration. On the amaZon/Esquire the source parameters for SpheriCal^®^‐ESI were: infusion rate, 1.0 μL/min; capillary voltage, 3500 V; endplate offset voltage, 500 V; nebulizer pressure, 0.1 Bar; dry gas flow rate, 4.5 L/min; 300°C. Ion guide parameters were automatically set using “smart parameter settings” (SPS) at the *m*/*z* value of the calibrant. For evaluation, spectra were recorded with SPS at *m*/*z* 250, 500, 1000, and 1500, and the most intense peak reported. Only peaks with *S*/*N* > 3 and confirmed charge state in all five repeats were reported. The trap parameters were: ion charge control (ICC), 100 000/20 000 (as the scan range was reduced during calibration, ICC was decreased to 5000 on the Esquire, and although the amaZon supposedly adapts ICC to the scan range, stepwise lowering of the ICC from 100 000 at S273–S1086 to 25 000 at S1716 for SpheriCal^®^‐ESI and from 100 000 at *m*/*z* 118–922 to 50 000 at *m*/*z* 1522 for ESI Tuning Mix was necessary to avoid overloading); scan delay, 0/5 ms; Gauss filter, 0.05 *m*/*z* units; maximum accumulation time, 50 ms. The parameters for CytC were the same except: infusion rate, 2 μL/min; capillary voltage, 4000 V; dry gas flow rate, 6 L/min. The parameters for the ESI Tuning Mix were the same as for SpheriCal^®^‐ESI except: infusion rate, 2 μL/min; capillary voltage, 4500 V; dry gas flow rate, 4 L/min; 180°C (these default values for the ESI Tuning Mix are notably different from the peptide analysis parameters, necessitating some delay to let the desolvation capillary cool). The parameters for PEG‐600 were the same as for SpheriCal^®^‐ESI except for infusion at 2 μL/min. The He buffer gas pressures of the traps were calibrated automatically and manually for the amaZon and the Esquire, respectively, within 2 months of the last measurements.

On the Quattro micro the source parameters for SpheriCal^®^‐ESI were: infusion rate, 10 μL/min; capillary voltage, 3800 V; cone voltage ramping, 50 V at *m*/*z* 273 to 150 V at *m*/*z* 1716; nebulizer gas flow rate, 200 L/h; desolvation gas flow rate, 600 L/h; 300°C; source temperature, 120°C; cone gas flow rate, 30 L/h; extractor voltage, 3 V. The quadrupole settings were: LM1 and LM2 resolution, 15; HM1 and HM2 resolution, 14; ion energy1, 0.5; ion energy2, 2; collision energy, 2; entrance, 50; exit, 50; multiplier, 650 V. The parameters for CytC were the same except for the cone voltage ramping (50 V at *m*/*z* 200 to 150 V at *m*/*z* 2000). The spray probes on all instruments were adjusted for low flow rates (capillaries highly extended).

The sample path was rinsed with 0.2 mL of MeOH before and after infusing SpheriCal^®^‐ESI, and with 0.2 mL of MeCN before and after CytC digest.

### 2.6 Evaluation of memory effects and contamination

Memory effect tests were performed on the amaZon in “Ultrascan” mode (32 500 *m*/*z* units/s), 20 scans per spectrum, SPS at *m*/*z* 900, for maximum sensitivity. SpheriCal^®^‐ESI was infused for 5 min, followed by immediate infusion of a blank, or by a rinse and then a blank. The first blank sample was a typical ESI peptide analysis solvent, for example as during a liquid chromatography (LC) analysis (the same as used for analysis of CytC): MeCN (60 vol%) and HCOOH (0.1 vol%) in water, 2 μL/min, source settings as used during CytC analysis. The second blank was an example of the nonpolar component of another LC gradient: 0.1 vol% HCOOH in MeOH, 2 μL/min, source settings as used for SpheriCal^®^‐ESI analysis. The third blank was the solvent for SpheriCal^®^‐ESI, NaHCOO (5 μg/mL) in MeOH, 1 μL/min, source settings as used during SpheriCal^®^‐ESI analysis, presumably giving the highest sensitivity for detecting any memory effects. Rinsing was performed manually with 0.2 mL of 1 vol% HCOOH in MeCN followed by 0.2 mL of water. A similar test was performed with PEG‐600, although the infusion time was limited to 1 min, rinsing was performed twice, and the last blank was pure MeOH.

To check for unwanted Na^+^‐adducts, SpheriCal^®^‐ESI was infused for 5 min directly followed by infusion of the CytC digest. The instrument was thoroughly rinsed with MeOH and water and the same CytC digest infused again for comparison. CytC was analyzed as in the calibration evaluation except using “Ultrascan” mode with 20 averages. A manual search for Na^+^‐adducts was performed for peaks that were identified in the CytC digest after SpheriCal^®^‐ESI calibration of the amaZon (35 *m*/*z* values corresponding to 18 peptides); however, neglecting mixed adducts.

### 2.7 MS^*n*^ analysis of SpheriCal^®^‐ESI and peptides

MS^*n*^ spectra of SpheriCal^®^‐ESI were recorded using the Esquire in “Normal resolution” mode (13 000 *m*/*z* units/s), scan, isolation, and fragmentation manually calibrated against SpheriCal^®^‐ESI. The settings were as during calibration evaluation but with ICC of 10 000 and SPS at the precursor *m*/*z* value. The isolation width was 4 *m*/*z* units, fragmentation width 10 *m*/*z* units, and fragmentation time 40–100 ms (longer times being beneficial for higher *m*/*z*). The scan range and fragmentation amplitude were adapted for each precursor. He buffer gas was used for collision‐induced dissociation (CID).

MS^2^ spectra on the Quattro micro were recorded after the standard automated calibration of static scan and MS2 scan. Settings were as during calibration evaluation but with: LM1 resolution, 13; HM1 resolution, 12; entrance, 5; exit, 18. The MS2 scan range and collision energy were adapted for each precursor. Ar gas was used for CID.

Intact CytC spectra and MS^*n*^ spectra for structure elucidation were recorded on the amaZon in “Enhanced resolution” mode (8100 *m*/*z* units/s), ICC of 200 000, scan calibrated against a solvent cluster at *m*/*z* 101 and S273–S1716, fragmentation against S273–S1716, and isolation against S273–S888. The MS^*n*^ settings were otherwise the same as for the Esquire, and source settings as during calibration evaluation. Amino acid sequences were manually assigned and checked against theoretical dissociation.^16^, ^17^

## 3 RESULTS AND DISCUSSION

### 3.1 SpheriCal^®^‐ESI performance

**FIGURE 1:**
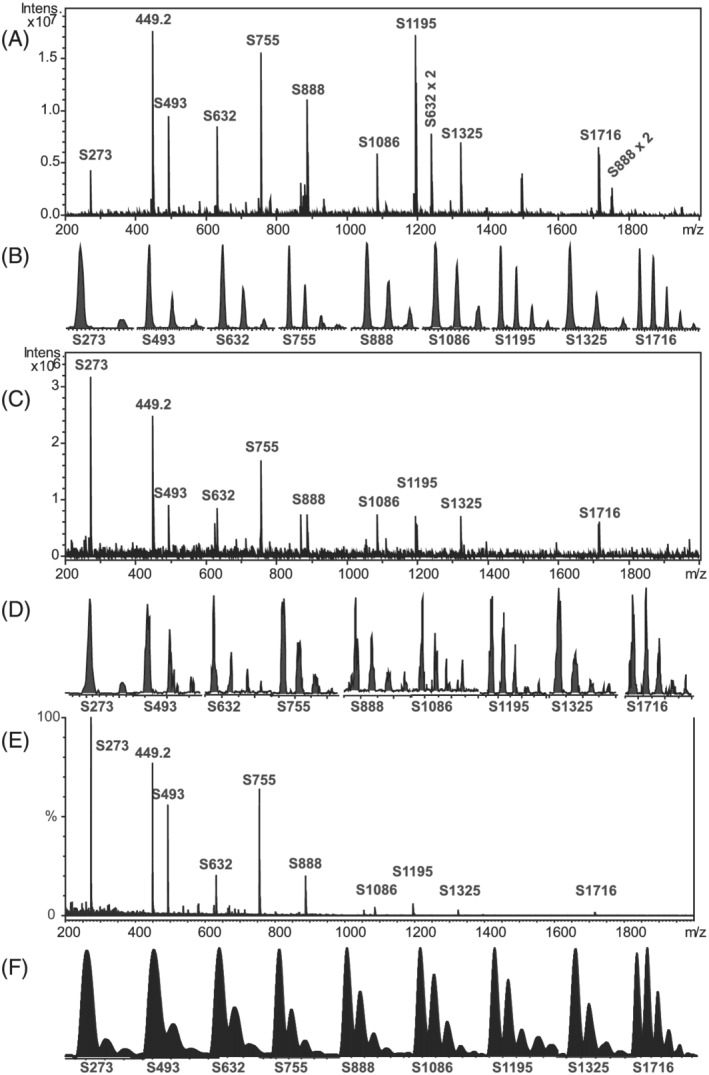
Mass spectra of SpheriCal^®^‐ESI. (A) Full scan on the amaZon ion trap, SPS at *m*/*z* 1500, 50 scans. Two dimers are also seen. (B) Peak shapes in (A). (C) Full scan on the Esquire ion trap, overlay of SPS at *m*/*z* 250, 500, 1000, and 1500, 20 scans. (D) Peak shapes in (C). (E) On the Quattro micro tandem quadrupole, three scans. (F) Peak shapes in (E)

As SpheriCal^®^‐ESI is intended for peptide analysis, care was taken to get good signals using similar or the same source parameters as used with peptides. The appearances of spectra and peak shapes for the three instruments are shown in Figure 1. Although not shown here, high performance was also obtained at both higher and lower capillary voltages, dry gas flow rates, and temperatures. Increased infusion rates gave more intense and more stable signals, especially on the Quattro micro. An intense contamination peak, *m*/*z* 449.2 (synthesis reactant; Table S1, supporting information), originated from this batch of S1716, but did not show any obvious detrimental effects. No signals from multiply charged dendrimers were seen, but two dimers were seen (confirmed by MS^2^), marked in Figure 1A. The Na^+^‐adducts were by far the most intense, but low‐intensity intermittent NH_4_^+^‐adducts (5 *m*/*z* units lighter) were sometimes detected, and, for S632 and S888, intermittent low‐intensity signals 6 *m*/*z* units lighter, probably Triton contamination.^18^ Intense NH_4_^+^‐adducts could be seen if samples containing NH_4_^+^ had been previously analyzed without sufficient rinsing afterwards. Currently, SpheriCal^®^‐ESI does not give any significant signals in negative ion mode.

Benchtop storage of the dried calibration mixtures showed no loss of performance during 2 months (Figure S1, supporting information) (the length of the test), but SpheriCal^®^‐ESI in MeOH should preferably be used the same day; on longer storage in solution the intensity of S1325 diminished (Figure S2 (top), supporting information). Dissolving in 20 vol% THF in MeOH gave similar performance to MeOH but a shelf life of at least four days on the benchtop (Figure S2 (bottom), supporting information).

Automated calibration tended to overload the ion traps at higher *m*/*z* regardless of the calibration solution used, and, due to the slow scan speed, was prohibitively slow on the Esquire (over 1 h compared with 10–15 min for manual). Therefore, manual calibration is always used for these instruments. During the manual calibration of the ion traps, lowering the ion count in the trap reduced peak distortion and improved accuracy. Using a substantially lowered ion count and averaging many more spectra during both calibration and scan may allow for slightly better accuracy than obtained here but becomes impractically slow for routine peptide analysis. Similar to averaging, lower resolution may improve accuracy and precision if smoother peaks are obtained.^19^

For the Quattro micro, it was noted that increasing the expected error of 1 to 2 *m*/*z* units could lead to erroneous calibration against the second isotope peak (the most intense) for S1716. Furthermore, a much faster scan (>2000 *m*/*z* units/s) could lead to missed calibration points in the higher *m*/*z* range (increasing the infusion rate would probably improve this).

It was later found that an intense solvent cluster, [(MeOH)_2_ + (H_2_O)_2_ + H]^+^ (confirmed by MS^*n*^), calculated *m*/*z* 101.080835, could be used to extend calibration towards lower *m*/*z*. This cluster was included in scan calibration of the “Enhanced resolution” mode of the amaZon for MS^*n*^ experiments.

### 3.2 Calibration accuracy and precision

Evaluation of the calibrant peaks themselves was straightforward. However, there was some ambiguity for the peptide peaks: the [M + H]^+^ ion of what was supposedly 102‐ATNE‐105, calculated *m*/*z* 434.19, showed an unexpectedly high *m*/*z* value, and the signal was removed from the comparison as the MS^2^ spectrum did not match that of the expected peptide (Figure S3 and Table S2, supporting information). 10‐IFVQKCAQCHTVEK‐23 (calculated *m*/*z* values of [M + H]^+^, [M + 2H]^2+^, and [M + 3H]^3+^ at 1633.82, 817.41, and 545.28) was distinguished from 15‐CAQCHTVEK‐23*[C_34_H_32_N_4_O_4_Fe(III)]^+^ (calculated *m*/*z* values of [M]^+^, [M + H]^2+^, and [M + 2H]^3+^ at 1633.61, 817.31, and 545.21) by MS^*n*^ (the latter was identified) (Figure S4 and Table S3, supporting information). The presence of heme B with Fe is confirmed because the main peak of intact CytC, [M + 15H]^16+^ at 773.47, deconvolutes to a molecular mass of 12 360.4 Da (calculated average 12 360.1 Da for uniprotID P00004, with removal of initial M, acetylation of 2‐G, and bound heme B, Fe(III)). The close *m*/*z* values of the two peptides above exemplifies how attempting to calibrate against protein digests can be precarious.

A similar *m*/*z* error over the whole range was expected as the instruments used for evaluation have constant bandwidth.^20^ Therefore, the evaluation is shown in *m*/*z* rather than ppm. Visual inspection of data from all three instruments showed that the *m*/*z* errors appeared reasonably normally distributed, and thus normal distribution was assumed for all statistics. The results of the statistical evaluation are presented in Table 2.

**TABLE 2:** Statistical evaluation of *m*/*z* errors on the three instruments

Sample	*m*/*z* error range	*m*/*z* error (average ± *σ*)	*P*_systematic‐error_
amaZon, SpheriCal			
SpheriCal	−0.03 to 0.04	0.01(0) ± 0.01(7)	>0.05
CytC	−0.06 to 0.05	0.00(2) ± 0.02(0)	>0.05
CytC 26 peaks of lowest *S*/*N*	−0.06 to 0.05	0.00(1) ± 0.02(1)	>0.05
CytC *S*/*N* >10	−0.04 to 0.05	0.00(3) ± 0.01(7)	>0.05
amaZon, Tuning Mix			
Tuning Mix	−0.09 to 0.07	−0.01(9) ± 0.03(7)	<0.05
CytC	−0.12 to 0.08	−0.00(6) ± 0.03(4)	<0.05
CytC ex. extrapol.	−0.12 to 0.08	−0.00(5) ± 0.03(3)	>0.05
CytC ex. extrapol. *S*/*N* > 10	−0.12 to 0.08	−0.00(5) ± 0.03(3)	>0.05
Esquire			
SpheriCal	−0.20 to 0.07	−0.03(2) ± 0.05(2)	<0.01
CytC	−0.28 to 0.07	−0.01(6) ± 0.05(3)	<0.01
Quattro micro			
SpheriCal	−0.08 to 0.00	−0.03(5) ± 0.01(7)	<0.01
CytC	−0.08 to 0.10	0.02(9) ± 0.03(6)	<0.01

**FIGURE 2:**
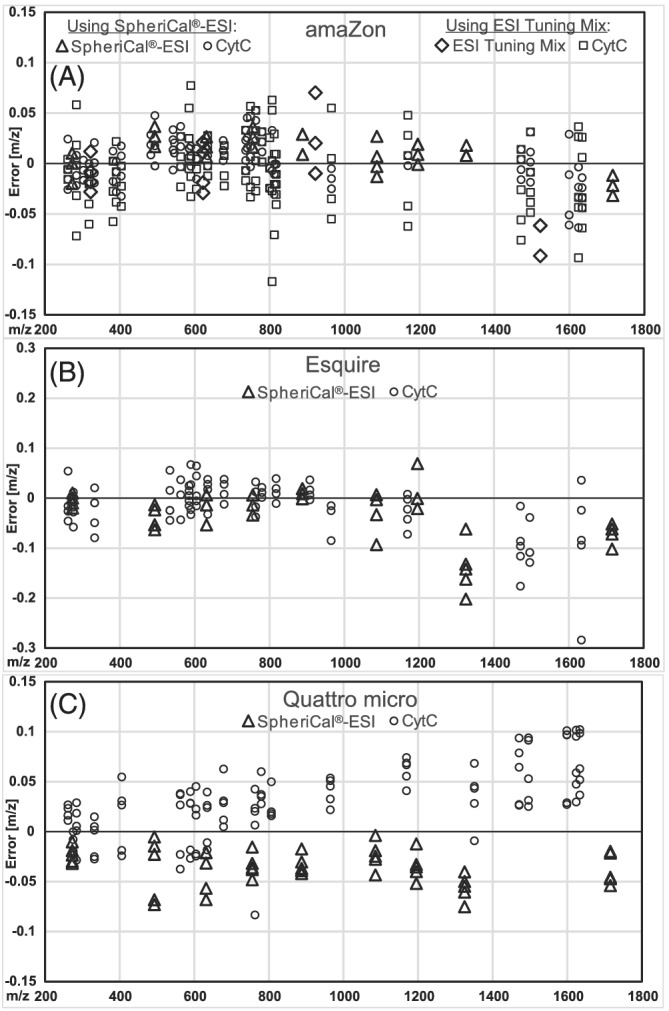
Evaluation of accuracy and precision of calibration (A) using SpheriCal^®^‐ESI or ESI Tuning Mix on the amaZon ion trap, (B) using SpheriCal^®^‐ESI on the Esquire ion trap (note the different scale), and (C) using SpheriCal^®^‐ESI on the Quattro micro tandem quadrupole. Errors shown in *m*/*z* for SpheriCal^®^‐ESI after calibration with SpheriCal^®^‐ESI (triangles), CytC digest after calibration with SpheriCal^®^‐ESI (circles), ESI Tuning Mix after calibration with ESI Tuning Mix (diamonds), and CytC digest after calibration with ESI Tuning Mix (squares)

From the evaluations of the calibrations (Figure 2), it is obvious that the amaZon had superior accuracy and precision. As a reference, the instrument manufacturer recommends recalibration if mass errors are greater than 0.15 Da (*m*/*z* units).

After calibration of the amaZon using SpheriCal^®^‐ESI, 35 *m*/*z* values corresponding to 18 peptides were found. An example spectrum of CytC can be seen in Figure S5 (supporting information).

After calibration of the amaZon using ESI Tuning Mix, only 26 *m*/*z* values, corresponding to 16 peptides, were found (this difference is discussed below). The ESI Tuning Mix evaluation had the small advantage of having helium pressure and detector response calibrated earlier the same day.

In comparing the performance of SpheriCal^®^‐ESI and Tuning Mix it was noted that both the average and the standard deviation of *m*/*z* errors were lower using SpheriCal^®^‐ESI than when using ESI Tuning Mix. Welsh's two‐tailed *t*‐test showed statistical significance for the difference in *m*/*z* average errors between calibration with SpheriCal^®^‐ESI and ESI Tuning Mix on the amaZon for both the calibrants themselves and the CytC digest (Table 3). The two‐tailed *F*‐test showed that the variances also were significantly different for the calibrants themselves and the CytC digest (Table 3).

**TABLE 3:** Comparison of *m*/*z* accuracy and precision between SpheriCal^®^‐ESI and Tuning Mix

SpheriCal^®^‐ESI versus Tuning Mix	*P*_*t*‐test_	*P*_*F*‐test_
Original data		
SpheriCal – SpheriCal versus Tuning Mix – Tuning Mix	0.03	3 × 10^–5^
SpheriCal – CytC versus Tuning Mix – CytC	0.01	4 × 10^–11^
Only 26 peaks and removed extrapolated peaks		
SpheriCal – CytC versus Tuning Mix – CytC	0.08	2 × 10^–6^
Removed extrapolated peaks and *S*/*N* < 10		
SpheriCal – CytC versus Tuning Mix – CytC	0.02	1 × 10^–12^

However, there were two important differences between the tests using CytC digest. First, ESI Tuning Mix was evaluated much later and only 26 *m*/*z* values corresponding to 16 peptides were found in the CytC digest. Similarly, looking at the first of the five repeats, the average *S*/*N* in the CytC digest was 112 for the SpheriCal^®^‐ESI evaluation and 57 for the ESI Tuning Mix evaluation. To prevent the higher *S*/*N* values from skewing the results in favor of SpheriCal^®^‐ESI, all peaks from the ESI Tuning Mix evaluation were compared with the 26 peaks of lowest *S*/*N* from the SpheriCal^®^‐ESI evaluation (Table 2). Second, for ESI Tuning Mix two high *m*/*z* peaks were outside the calibrated range (*m*/*z* > 1522) (one peptide was outside the SpheriCal^®^‐ESI range, but only by 12 *m*/*z* units). These two peaks did not show significantly different average error or variance from the other peaks (*P*_one‐tail_ = 0.26 and 0.17). Nevertheless, removing them from the dataset removed the systematic error (Table 2). Importantly, while the performance still appeared worse than for the 26 lowest *S*/*N* peaks from using SpheriCal^®^‐ESI, the difference in average error was no longer statistically significant, although the difference in variance still was (Table 3). Another way of removing these biases is to exclude all peaks with *S*/*N* < 10 (4 for SpheriCal^®^‐ESI and 3 for ESI Tuning Mix), and the two extrapolated peaks (Table 2). The difference in average error and the variance were then significant (Table 3).

A higher accuracy and/or precision obtained for SpheriCal^®^‐ESI could be explained by there being more calibration points in the range of interest (nine compared with five), and that the intense peaks are in the range where most peptides were found (compare Figures 1A and 2A). In contrast, for ESI Tuning Mix, *m*/*z* 622 had the lowest relative intensity (of *m*/*z* 118, 322, 622, 922, and 1522). It should be mentioned that there is a possibility of subconscious human bias during the manual calibration. Although not evaluated here, Pierce™ LTQ ESI Positive Ion Calibration Solution showed only one, relatively low‐intensity, peak (*m*/*z* 524) between *m*/*z* 200 and 900 and was not expected to perform better than ESI Tuning Mix. PEG‐600 has many narrowly spaced peaks in this region but has limited range and severe memory effects (as discussed in section 3.3).

For the Esquire, 19 *m*/*z* values, corresponding to 17 peptides, were found (Figure 2B). The systematic error (Table 2) can be explained by the trap being slightly overloaded during calibration, especially at higher *m*/*z*, giving systematic errors towards lower *m*/*z*. A possible explanation for the poor precision is the slow scan speed, allowing for fewer scans per spectrum, leading to the peak splitting seen in Figure 1D (and Figure S2 (bottom), supporting information) and spectrum‐to‐spectrum variation. In addition, extensive unauthorized trap service may have negatively affected performance.

On the Quattro micro, 21 *m*/*z* values, corresponding to 21 peptides, were found (Figure 2C). The reason that the systematic error (Table 2) was to lower *m*/*z* for SpheriCal^®^‐ESI but to higher *m*/*z* for CytC is unknown, but is perhaps related to putting the instrument in standby before measuring CytC. Possibly picking a higher centroid will improve the accuracy at low resolution.^21^

### 3.3 Memory effects and contamination

**FIGURE 3:**
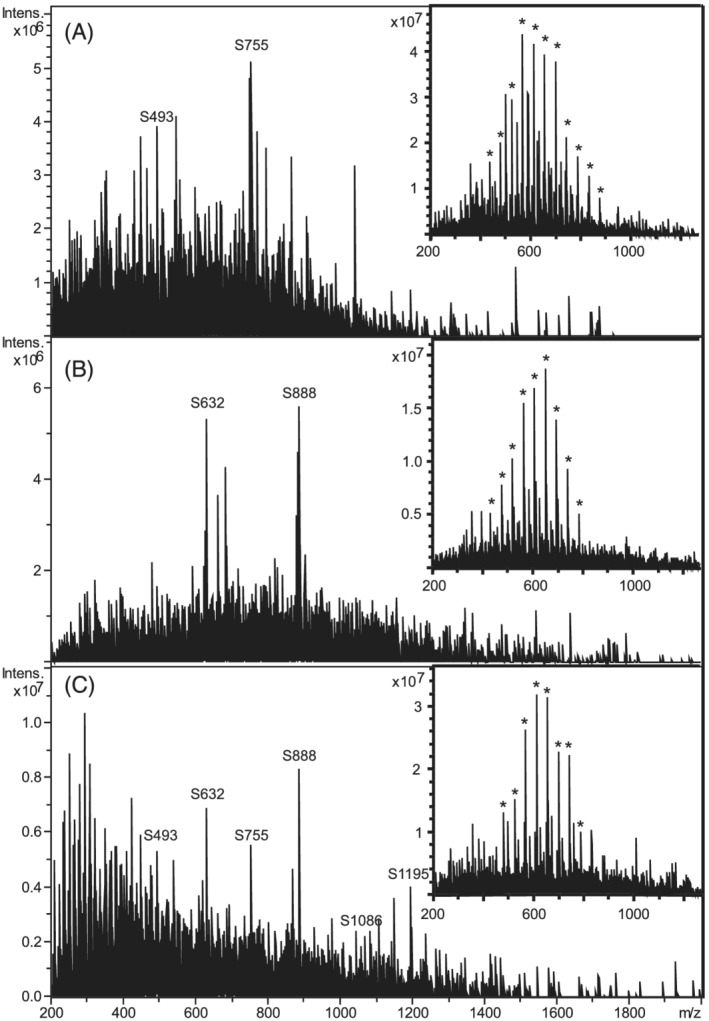
Memory effects of SpheriCal^®^‐ESI in full scan on the amaZon in “Ultrascan” mode, 20 scans, after 5 min infusion of SpheriCal^®^‐ESI. (A) Peptide analysis blank (MeCN (60 vol%), HCOOH (0.1 vol%) in water) directly after SpheriCal^®^‐ESI. Less than 1 μL infused. (B) Nonpolar mobile phase blank (0.1 vol% HCOOH in MeOH), directly after SpheriCal^®^‐ESI. 50 μL infused. (C) SpheriCal^®^‐ESI blank (NaHCOO (5 μg/mL) in MeOH) after rinsing with 0.2 mL of HCOOH (1 vol%) in MeCN and 0.2 mL of water. 1 μL infused. The insets show the memory effects of 50 μg/mL PEG‐600 under the same conditions except 1 min infusion, and two rinses and a MeOH blank for (C). Asterisks mark the main peaks of PEG‐600 Na^+^‐adducts

Directly after infusion of SpheriCal^®^‐ESI for 5 min, infusion of a typical ESI peptide solvent blank, MeCN (60 vol%) and HCOOH (0.1 vol%) in water, reduced SpheriCal^®^‐ESI peaks to baseline levels after less than 1 μL had been infused, as shown in Figure 3A. This makes the current SpheriCal^®^‐ESI system unsuitable for internal calibration during peptide analysis. However, with a blank of HCOOH (0.1 vol%) in MeOH two SpheriCal^®^‐ESI peaks were visible at low intensity even after ca 50 μL had been infused (Figure 3B). A rinse with 0.2 mL of HCOOH (1 vol%) in MeCN followed by 0.2 mL of water lowered the SpheriCal^®^‐ESI peaks close to baseline even when using a SpheriCal^®^‐ESI blank (5 μg/mL NaHCOO in MeOH) (Figure 3C). Initial tests showed that some SpheriCal^®^‐ESI remained (as seen when injecting the SpheriCal^®^‐ESI blank) when rinsing with only 0.2 mL of either pure MeOH, MeCN, or water, but no traces of SpheriCal^®^‐ESI were detected during analysis of the CytC digest. Thus, SpheriCal^®^‐ESI showed very minimal memory effects under peptide analysis conditions.

As expected, PEG‐600 at 50 μg/mL showed severe memory effects (insets in Figure 3) which could only be completely removed by rinsing with CH_2_Cl_2_ (not shown). This was also the case for 10 μg/mL. No severe memory effects from either ESI Tuning Mix or Pierce™ LTQ ESI Positive Ion Calibration Solution were noted.

Na^+^‐adducts were negligible in the analyzed CytC spectra. For the reference spectra (after thorough rinsing), two Na^+^‐adducts were found at *m*/*z* 440 and 828 with *S*/*N* values of 18 (23 for the corresponding protonated peptide) and 5 (65), respectively. In the spectra recorded immediately after SpheriCal^®^‐ESI both these peptides were seen with *S*/*N* values of 32 (58) and 5 (66), respectively, but one additional Na^+^‐adduct was potentially detected at *m*/*z* 403 with *S*/*N* of 4 (11) (the corresponding protonated peptide has *S*/*N* of 5 in the reference spectrum). In this experiment it was also confirmed that no SpheriCal^®^‐ESI peaks were detectable in the spectrum of CytC even without rinsing.

### 3.4 MS^*n*^ spectra

MS^*n*^ analysis showed that several intense product ions were common to CID in the tandem quadrupole and the ion trap, allowing calibration of MS^*n*^ using product ions (although this was not required for the instruments used), or checking instrument MS^*n*^ performance. However, it was also noted that some high‐intensity product ions were unique to each instrument. Table S4 (supporting information) presents selected common intense product ions for which plausible theoretical dissociation pathways were found.

## 4 CONCLUSIONS

SpheriCal^®^‐ESI enabled facile external calibration with the same or similar source settings as used in peptide analysis, also at low infusion rates, even with demanding scan settings using an obsolete instrument. The accuracies were as expected for the instrument types under typical use^20^ and SpheriCal^®^‐ESI calibration appeared more precise than and at least as accurate as the recommended calibration mix, ESI Tuning Mix. Importantly, SpheriCal^®^‐ESI showed negligible memory effects during peptide analysis conditions, eliminating one of the biggest issues of many existing calibrants. Furthermore, no Na^+^ contamination was seen. Lastly, the reproducible and predictable dissociation of the dendrimers allowed structure confirmation and evaluation of instrument MS^*n*^ performance, and may also allow MS^*n*^ calibration where necessary.

5

### PEER REVIEW

The peer review history for this article is available at https://publons.com/publon/10.1002/rcm.9035.

## Supporting information


**Table S1.** MS^n^ analysis for the identification of the contaminant at *m/z* 449.2: bis‐MPA benzylidene anhydride, used in the synthesis of S1716.
**Figure S1**. Shelf‐life of dry SpheriCal®‐ESI. Top: freshly prepared. Bottom: after storing the dried vial on the benchtop for 2 months 13 days. Note the different scales. Spectra were obtained on the amaZon in maximum resolution mode, at SPS 1500, 50 scans/spectrum.
**Figure S2**. Shelf‐life of SpheriCal®‐ESI in solution. Top: SpheriCal®‐ESI in MeOH after 1 day storage on benchtop. Bottom: SpheriCal®‐ESI in 20 vol% THF in MeOH after 4 days storage on benchtop. Only the signal for S1325 is shown as the other dendrimers showed little signal loss on storage. Spectra were obtained on the esquire in maximum resolution mode, at SPS 1500, 10 scans/spectrum.
**Figure S3**. MS^2^ spectrum of *m/z* 434, indicating that what was supposedly 102‐ATNE‐105 is something else as the theoretical dissociation of ATNE does not fit the spectrum (see Supplementary Table 2).
**Table S2**. MS^2^ analysis for the identification of the compound at *m/z* 434 (Supplementary Figure 3). The major product peak at *m/z* 262 has not been identified, but the peptide EGTK is otherwise a good fit to the spectrum (full b‐series).
**Figure S4**. MS^2^ spectrum of *m/z* 817, to distinguish 10‐IFVQKCAQCHTVEK‐23 from 15‐CAQCHTVEK‐23*[C_34_H_32_N_4_O_4_Fe(III)]+. (See Supplementary Table 3).
**Table S3**. MS^2^ analysis of *m/z* 817 (Supplementary Figure 4), showing that the peak belongs to 15‐CAQCHTVEK‐23*[C_34_H_32_N_4_O_4_Fe(III)]+. Although not shown here, the peaks containing Fe (III) clearly showed the isotope pattern expected from Fe.
**Table S4**. Selected intense product ions of SpheriCal®‐ESI dendrimers common to CID in both the Esquire ion trap (He) and Quattro micro tandem quadrupole (Ar). Both instruments produced unique high intensity product ions not included in the table. Due to insufficient instrument accuracy for assigning elemental compositions product ions were tentatively identified by mass agreement with theoretical dissociation pathways and additional MS^n^ experiments. The most certain product ions are in bold (high mass agreement on both instruments and highly plausible dissociation pathway). *Not seen in ion trap due to *m/z* cut‐off.
**Figure S5**. MS spectrum of the CytC digest. The spectrum was obtained on the amaZon in maximum resolution mode, at SPS 1500, 50 scans/spectrum.

**Data S1.** Supporting Information
