# Supplementary material for: SpheriCal®‐ESI: A dendrimer‐based nine‐point calibration solution ranging from m/z 273 to 1716 for electrospray ionization mass spectrometry peptide analysis
Source: Rapid Commun Mass Spectrom. 2021 Jan 21;35(5):e9035. doi: 10.1002/rcm.9035 (PMC7900979; doi:10.1002/rcm.9035)
Supplement: Supplementary file 1 — Table S1. MSn analysis for the identification of the contaminant at m/z 449.2: bis‐MPA benzylidene anhydride, used in the synthesis of S1716. Figure S1. Shelf‐life of dry SpheriCal®‐ESI. Top: freshly prepared. Bottom: after storing the dried vial on the benchtop for 2 months 13 days. Note the different scales. Spectra were obtained on the amaZon in maximum resolution mode, at SPS 1500, 50 scans/spectrum. Figure S2. Shelf‐life of SpheriCal®‐ESI in solution. Top: SpheriCal®‐ESI in MeOH after 1 day storage on benchtop. Bottom: SpheriCal®‐ESI in 20 vol% THF in MeOH after 4 days storage on benchtop. Only the signal for S1325 is shown as the other dendrimers showed little signal loss on storage. Spectra were obtained on the esquire in maximum resolution mode, at SPS 1500, 10 scans/spectrum. Figure S3. MS2 spectrum of m/z 434, indicating that what was supposedly 102‐ATNE‐105 is something else as the theoretical dissociation of ATNE does not fit the spectrum (see Supplementary Table 2). Table S2. MS2 analysis for the identification of the compound at m/z 434 (Supplementary Figure 3). The major product peak at m/z 262 has not been identified, but the peptide EGTK is otherwise a good fit to the spectrum (full b‐series). Figure S4. MS2 spectrum of m/z 817, to distinguish 10‐IFVQKCAQCHTVEK‐23 from 15‐CAQCHTVEK‐23*[C34H32N4O4Fe(III)]+. (See Supplementary Table 3). Table S3. MS2 analysis of m/z 817 (Supplementary Figure 4), showing that the peak belongs to 15‐CAQCHTVEK‐23*[C34H32N4O4Fe(III)]+. Although not shown here, the peaks containing Fe (III) clearly showed the isotope pattern expected from Fe. Table S4. Selected intense product ions of SpheriCal®‐ESI dendrimers common to CID in both the Esquire ion trap (He) and Quattro micro tandem quadrupole (Ar). Both instruments produced unique high intensity product ions not included in the table. Due to insufficient instrument accuracy for assigning elemental compositions product ions were tentatively identified by mass agreement with [file RCM-35-e9035-s001.pdf]

SpheriCal®-ESI Supplementary Figures and Tables

Supplementary Table 1. MS^n analysis for the identification of the contaminant at *m/z* 449.2: bis-MPA benzylidene anhydride, used in the synthesis of S1716.

Precursor	Product ion	Product ion *m/z*	Trap MS^n	MS^2 CID amplitude [V]	MS^3 precursor (CID amplitude [V])
Contaminant 449.2 [$C_{24}H_{26}O_7Na^+$], *m/z* 449.157074	[$C_{12}H_{13}O_3$]$^+$	205.085921	2	1.2-1.5	
	[$C_{12}H_{14}O_4Na$]$^+$	245.078430	2	1.2-1.5	
	[$C_{15}H_{16}O_5Na$]$^+$	299.088994	2; 3	1.2-1.5	343 (1.0)
	[$C_{17}H_{20}O_6Na$]$^+$	343.115209	2	1.2-1.5	

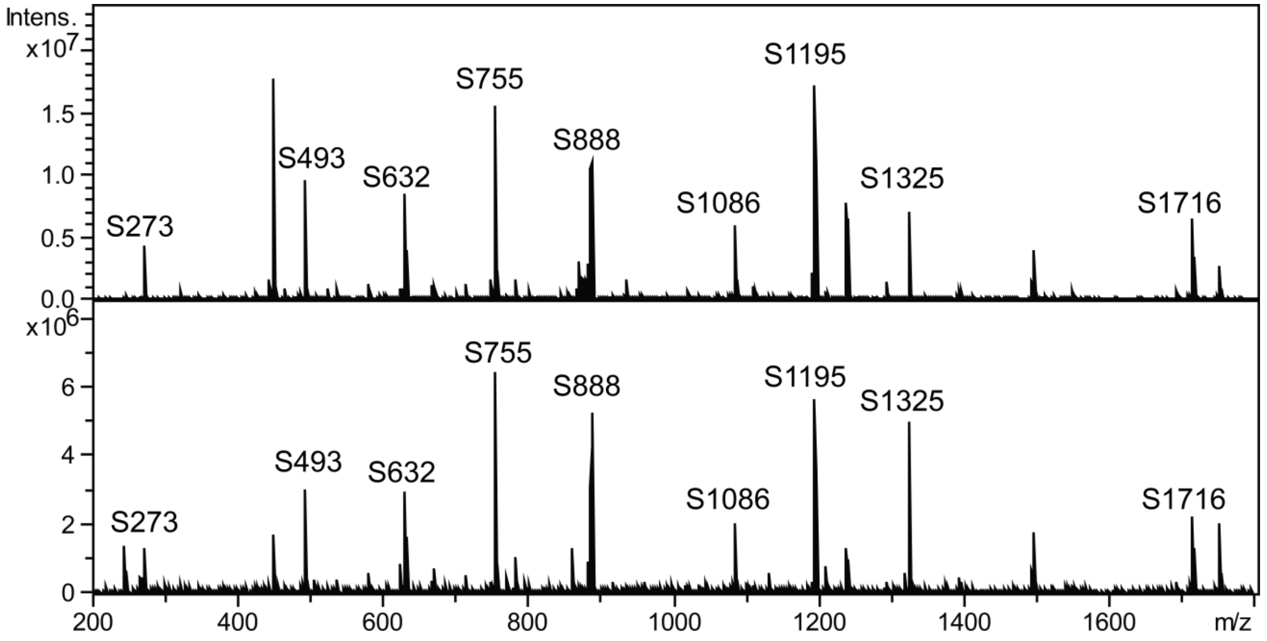

Supplementary Figure 1. Shelf-life of dry SpheriCal®-ESI. Top: freshly prepared. Bottom: after storing the dried vial on the benchtop for 2 months 13 days. Note the different scales. Spectra were obtained on the amaZon in maximum resolution mode, at SPS 1500, 50 scans/spectrum.

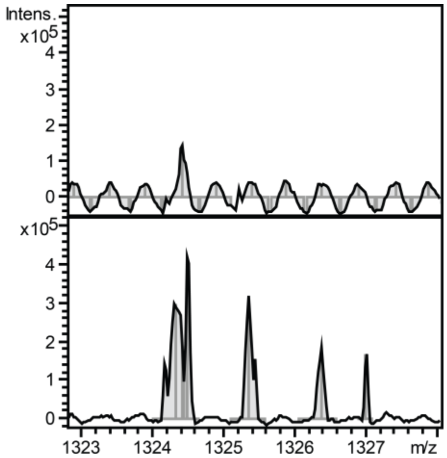

Supplementary Figure 2. Shelf-life of SpheriCal®-ESI in solution. Top: SpheriCal®-ESI in MeOH after 1 day storage on benchtop. Bottom: SpheriCal®-ESI in 20 vol% THF in MeOH after 4 days storage on benchtop. Only the signal for S1325 is shown as the other dendrimers showed little signal loss on storage. Spectra were obtained on the esquire in maximum resolution mode, at SPS 1500, 10 scans/spectrum.

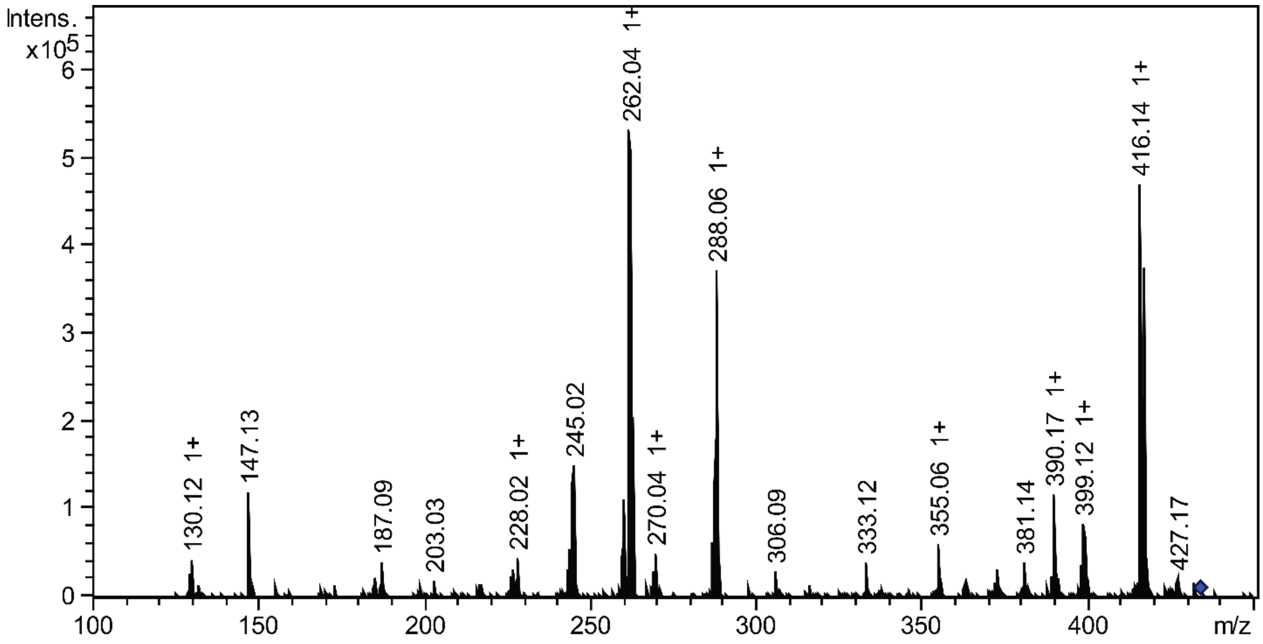

Supplementary Figure 3. MS^2 spectrum of *m/z* 434, indicating that what was supposedly 102-ATNE-105 is something else as the theoretical dissociation of ATNE does not fit the spectrum (see Supplementary Table 2).

Supplementary Table 2. MS^2 analysis for the identification of the compound at *m/z* 434 (Supplementary Figure 3). The major product peak at *m/z* 262 has not been identified, but the peptide EGTK is otherwise a good fit to the spectrum (full b-series).

Precursor	Product ion	Product ion *m/z*	MS^2 CID amplitude [V]
Unknown peptide, possibly [EGT[**K**/Q] + H]$^+$ *m/z* **434.224539**/434.188154	[E – H_2O + H]$^+$	130.049870	ca 1
	[[**K**/Q] + H]$^+$ / [E – HO + NH_2 + H]$^+$	**147.112804**/147.076419 / 147.076419	ca 1
	[EG - H_2O + H]$^+$	187.071333	ca 1
	[EGT – H_2O + H]$^+$	288.119012	ca 1
	[EGTK – H_2O + H]$^+$	416.213975	ca 1

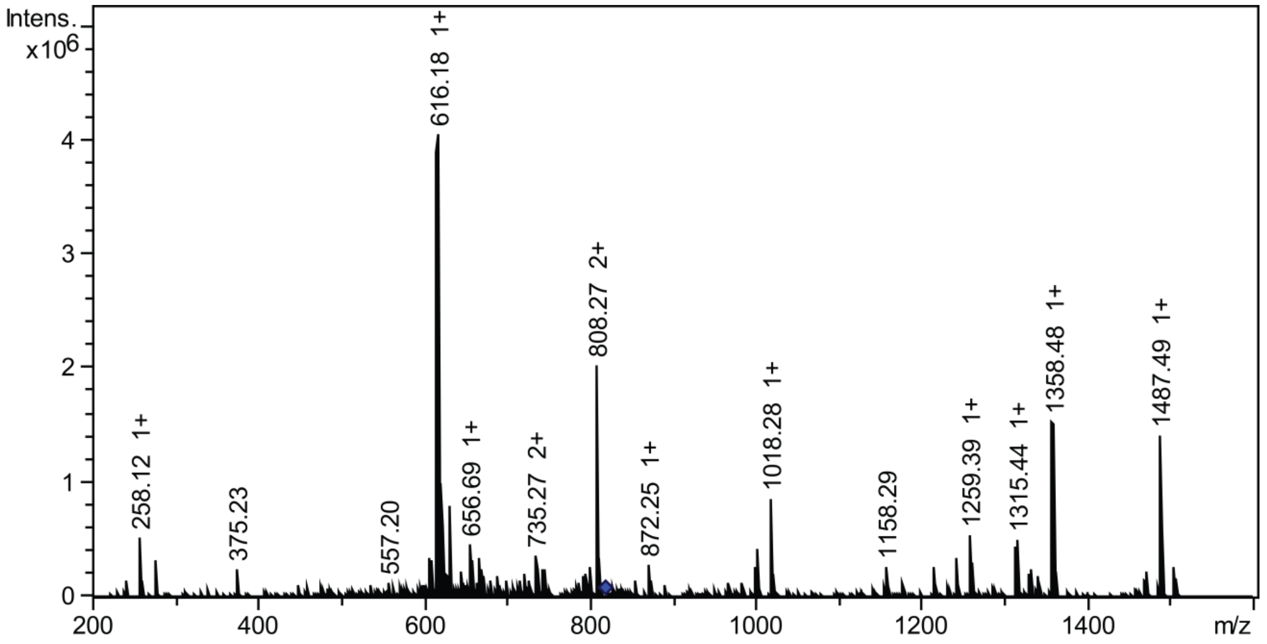

Supplementary Figure 4. MS^2 spectrum of *m/z* 817, to distinguish 10-IFVQKCAQCHTVEK-23 from 15-CAQCHTVEK-23*[$C_{34}H_{32}N_4O_4Fe(III)$]$^+$. (See Supplementary Table 3).

Supplementary Table 3. MS^2 analysis of *m/z* 817 (Supplementary Figure 4), showing that the peak belongs to 15-CAQCHTVEK-23*[$C_{34}H_{32}N_4O_4Fe(III)$]$^+$. Although not shown here, the peaks containing Fe(III) clearly showed the isotope pattern expected from Fe.

Precursor	Product ion	Product ion *m/z*	MS^2 CID amplitude [V]
[15-CAQCHTVEK-23*[$C_{34}H_{32}N_4O_4Fe(III)$]$^+$ + H]$^{2+}$ *m/z* 817.310603	Heme B ([$C_{34}H_{32}N_4O_4Fe(III)$]$^+$)	616.176743	ca 1
	[15-CAQCHTVEK-23 + H]$^+$	1018.444463	ca 1
	[15-CAQCHTV-21*[$C_{34}H_{32}N_4O_4Fe(III)$]$^+$ - H_2O]$^+$	1358.465808	ca 1
	[15-CAQCHTVE-22*[$C_{34}H_{32}N_4O_4Fe(III)$]$^+$ - H_2O]$^+$	1487.508401	ca 1

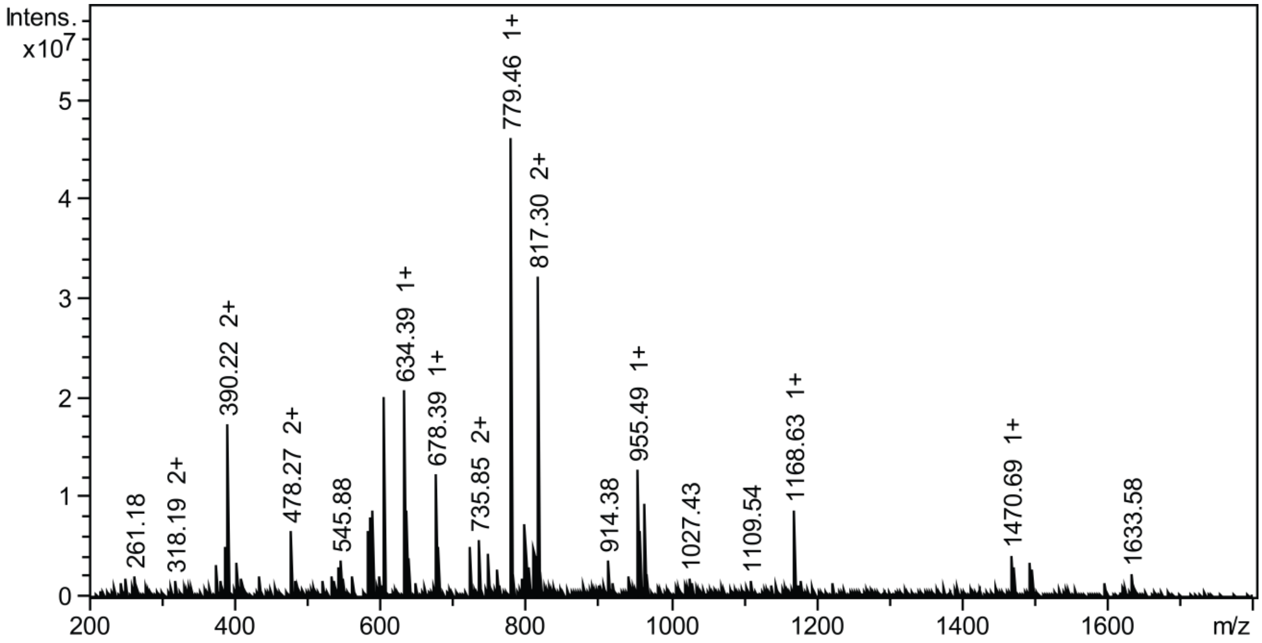

Supplementary Figure 5. MS spectrum of the CytC digest. The spectrum was obtained on the amaZon in maximum resolution mode, at SPS 1500, 50 scans/spectrum.

Supplementary Table 4. Selected intense product ions of SpheriCal®-ESI dendrimers common to CID in both the Esquire ion trap (He) and Quattro micro tandem quadrupole (Ar). Both instruments produced unique high intensity product ions not included in the table. Due to insufficient instrument accuracy for assigning elemental compositions product ions were tentatively identified by mass agreement with theoretical dissociation pathways and additional MS^n experiments. The most certain product ions are in bold (high mass agreement on both instruments and highly plausible dissociation pathway). *Not seen in ion trap due to *m/z* cut-off.

Precursor	Product ion	Product ion *m/z*	Quattro micro CID energy [eV]	Trap MS^n	MS^2 CID amplitude [V]	MS^3 precursor (CID amplitude [V])	MS^4 precursor (CID amplitude [V])
S273	$[C_5H_8O_3Na]^+$	139.036565	17	2; 3	1.2-1.3	245 (0.7)	
	$[C_{12}H_{13}O_3]^+$	205.085921	17	2	1.2-1.3		
	$[C_{12}H_{14}O_4Na]^+$	245.078430	17-22	2	1.2-1.3		
S493	$[C_7H_{11}O_2]^+$	127.075356	33-40	3		249 (0.9)	
	$[C_7H_{10}O_2Na]^+$	149.057300	33-40	2; 3	1.4	249 (0.9)	
	$[C_{12}H_{18}O_4Na]^+$	249.109730	33-40	2	1.4		
S632	$[C_6H_7O]^+$	95.049141	40	3		325 (1.3-1.4)	
	$[C_7H_9O]^+$	109.064791	40	3		325 (1.3-1.4)	
	$[C_8H_{11}O]^+$	123.080441	40	3		325 (1.3-1.4)	
	$[C_9H_{13}O]^+$	137.096091	40	3		325 (1.3-1.4)	
	$[C_{18}H_{35}O]^+$	267.268242	30	3		325 (1.3-1.4)	
	$[C_{21}H_{41}O_2]^+$	325.310107	30-40	2	0.9		
S755	$[C_{12}H_{13}O_3]^+$	205.085921	45	3; 4		375 (0.8)	375 (0.8)
	$[C_{13}H_{19}O_4]^+$	239.127786	45-55	2; 3; 4	1.2	375 (0.8); 511 (0.8)	375 (0.8)
	$[C_{21}H_{27}O_6]^+$	375.180215	45-55	2; 3	1.2	511	
	$[C_{29}H_{35}O_8]^+$	511.232645	45	2	1.2		
	$[C_{33}H_{40}O_{10}Na]^+$	619.251368	45-55	2	1.2		
S888	$[C_{29}H_{55}O_5]^+$	483.404401	50	3		666 (1.0)	
	$[C_{41}H_{77}O_6]^+$	665.571467	40-50	2	1.2		
	$[C_{41}H_{76}O_6Na]^+$	687.553411	40-50	2	1.2		
S1086	$[C_{29}H_{53}O_5]^+$	481.388751	45-50	2; 3	1.5	886 (1.0)	
	$[C_{41}H_{74}O_6Na]^+$	685.537761	45-50	2; 3	1.5	886 (1.0)	
	$[C_{53}H_{98}O_8Na]^+$	885.715391	45-50	2	1.5		
S1195	$[C_{12}H_{13}O_3]^+$	205.085921	55	3		449 (1.2); 679 (0.7)	
	$[C_{29}H_{32}O_9Na]^+$	547.193853	55-60	2; 3	1.6	973 (1.1)	
	$[C_{43}H_{52}O_{14}Na]^+$	815.324927	55	2	1.6		
	$[C_{53}H_{58}O_{16}Na]^+$	973.361706	55	2	1.6		
S1325	$[C_7H_4IO]^+$	230.930138	35-40	-*			
	$[C_{33}H_{23}I_4O_8]^+$	1054.756632	35-40	2	1.1		
S1716	$[C_{12}H_{13}O_3]^+$	205.085921	105	3		525 (1.0)	
	$[C_{22}H_{27}O_8]^+$	419.170044	105-115	3		525 (1.0); 1151 (1.3)	
	$[C_{80}H_{96}O_{27}Na]^+$	1511.603119	105	2	1.7		
	$[C_{84}H_{100}O_{28}Na]^+$	1579.629333	105-120	2	1.7		
