# Supplementary material for: SpheriCal®‐ESI: A dendrimer‐based nine‐point calibration solution ranging from m/z 273 to 1716 for electrospray ionization mass spectrometry peptide analysis
Source: Rapid Commun Mass Spectrom. 2021 Jan 21;35(5):e9035. doi: 10.1002/rcm.9035 (PMC7900979; doi:10.1002/rcm.9035)
Supplement: Supplementary file 2 — Data S1. Supporting Information [file RCM-35-e9035-s002.pdf]

SpheriCal®-ESI dendrimer structures

S273

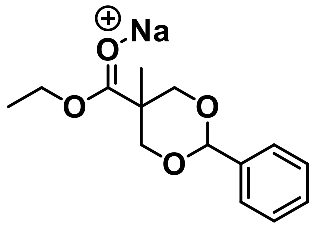

Chemical Formula: $C_{14}H_{18}NaO_4^+$
Exact Mass: 273.10973
Molecular Weight: 273.28322
m/z: 273.10973 (100.0%), 274.11308 (15.1%), 275.11644 (1.1%)

SMILES:
CC1(COC(C2=CC=CC=C2)OC1)/C(OCC)=[O+]\[Na]

m/z: **273.109730**

S493

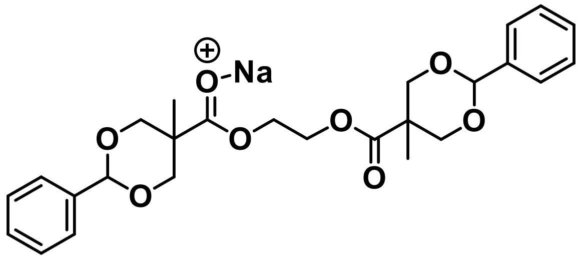

Chemical Formula: $C_{26}H_{30}NaO_8^+$
Exact Mass: 493.18329
Molecular Weight: 493.50722
m/z: 493.18329 (100.0%), 494.18664 (28.1%), 495.19000 (3.8%), 495.18753 (1.6%)

SMILES:
O=C(C1(C)COC(C2=CC=CC=C2)OC1)OCCO/C(C3(C)COC(C4=CC=CC=C4)OC3)=[O+]\[Na]

m/z: **493.183289**

S632

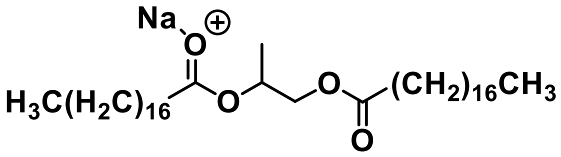

Chemical Formula: $C_{39}H_{76}NaO_4^+$
Exact Mass: 631.56358
Molecular Weight: 632.02222
m/z: 631.56358 (100.0%), 632.56694 (42.2%), 633.57029 (8.7%), 634.57365 (1.2%)

SMILES:
O=C(CCCCCCCCCCCCCCCCC)OCC(C)O/C(CCCCCCCCCCCCCCCCC)=[O+]/[Na]

***m/z:* 631.563582**

S755

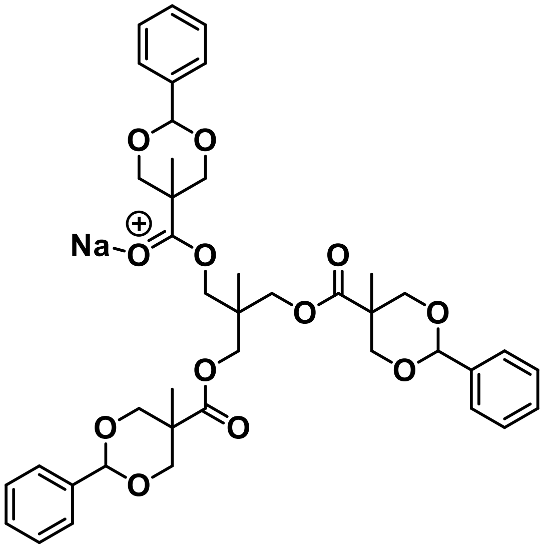

Chemical Formula: $C_{41}H_{48}NaO_{12}^+$
Exact Mass: 755.30380
Molecular Weight: 755.81222
m/z: 755.30380 (100.0%), 756.30715 (44.3%), 757.31051 (9.6%), 757.30804 (2.5%), 758.31386 (1.3%), 758.31140 (1.1%)

SMILES:
CC(COC(C1(C)COC(C2=CC=CC=C2)OC1)=O)(CO/C(C3(C)COC(C4=CC=CC=C4)OC3)=[O+]/[Na])COC(C5(C)COC(C6=CC=CC=C6)OC5)=O

***m/z:* 755.303798**

S888

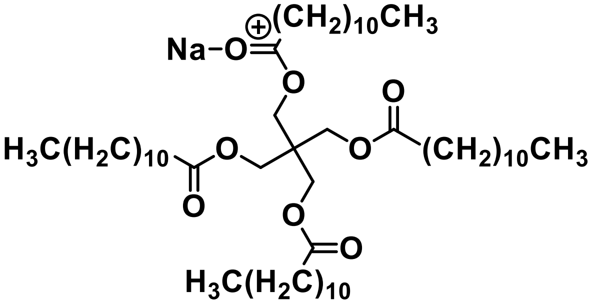

Chemical Formula: $C_{53}H_{100}NaO_8^+$
Exact Mass: 887.73104
Molecular Weight: 888.36422
m/z: 887.73104 (100.0%), 888.73439 (57.3%), 889.73775 (16.1%), 890.74110 (3.0%), 889.73529 (1.6%), 888.73732 (1.2%)

SMILES:
O=C(CCCCCCCCCCC)OCC(COC(CCCCCCCCCCC)=O)(COC(CCCCCCCCCCC)=[O+][Na])COC(CCCCCCCCCCC)=O

***m/z:* 887.731041**

S1086

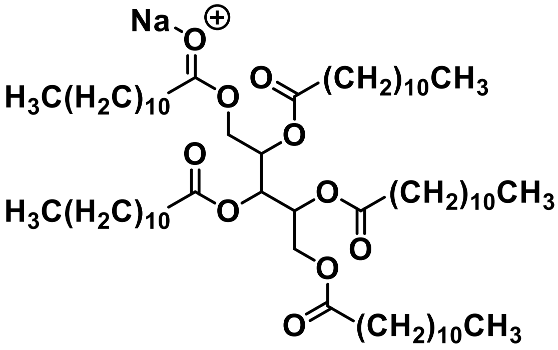

Chemical Formula: $C_{65}H_{122}NaO_{10}^+$
Exact Mass: 1085.89302
Molecular Weight: 1086.67022
m/z: 1085.89302 (100.0%), 1086.89637 (70.3%), 1087.89973 (24.3%), 1088.90308 (5.5%), 1087.89727 (2.1%), 1088.90062 (1.4%), 1086.89930 (1.4%), 1087.90265 (1.0%)

SMILES:
O=C(CCCCCCCCCCC)OC(C(CO/C(CCCCCCCCCCC)=[O+]/[Na])OC(CCCCCCCCCCC)=O)C(OC(CCCCCCCCCCC)=O
)COC(CCCCCCCCCCC)=O

***m/z:* 1085.893021**

S1195

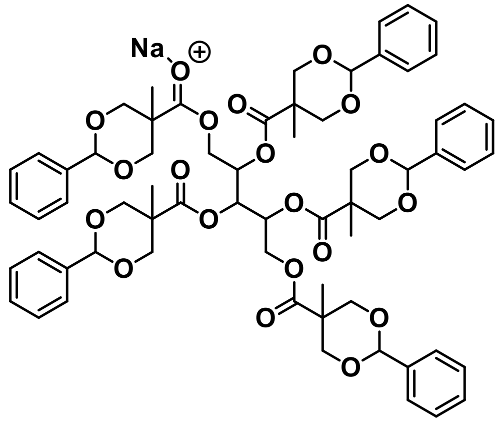

Chemical Formula: $C_{65}H_{72}NaO_{20}^{+}$
Exact Mass: 1195.45092
Molecular Weight: 1196.26022
m/z: 1195.45091 (100.0%), 1196.45427 (70.3%), 1197.45762 (24.3%), 1198.46098 (5.5%), 1197.45516 (4.1%), 1198.45852 (2.9%), 1199.46187 (1.0%)

SMILES:
O=C(C1(C)COC(C2=CC=CC=C2)OC1)OC(C(CO/C(C3(C)COC(C4=CC=CC=C4)OC3)=[O+]/[Na])OC(C5(C)COC(C6=CC=CC=C6)OC5)=O)C(OC(C7(C)COC(C8=CC=CC=C8)OC7)=O)COC(C9(C)COC(C%10=CC=CC=C%10)OC9)=O

m/z: **1195.450915**

S1325

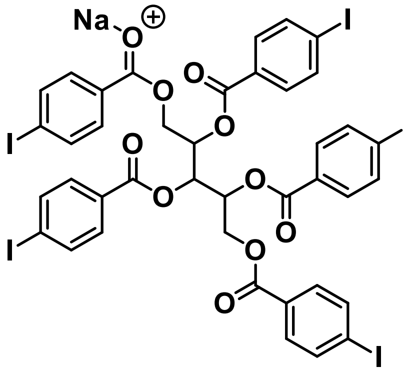

Chemical Formula: $C_{40}H_{27}I_5NaO_{10}^{+}$
Exact Mass: 1324.67198
Molecular Weight: 1325.15757
m/z: 1324.67198 (100.0%), 1325.67534 (43.3%), 1326.67869 (9.1%), 1326.67623 (2.1%), 1327.68205 (1.3%)

SMILES:
O=C(C1=CC=C(I)C=C1)OC(C(CO/C(C2=CC=C(I)C=C2)=[O+]/[Na])OC(C3=CC=C(I)C=C3)=O)C(OC(C4=CC=C(I)C=C4)=O)COC(C5=CC=C(I)C=C5)=O

m/z: **1324.672003***

S1716

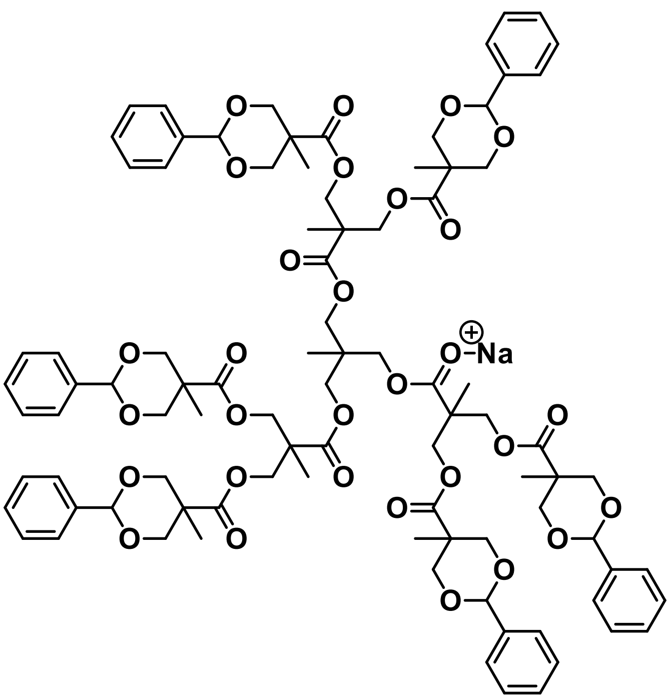

Chemical Formula: $C_{92}H_{108}NaO_{30}^{+}$
Exact Mass: 1715.68176
Molecular Weight: 1716.83522
m/z: 1715.68176 (100.0%), 1716.68512 (99.5%), 1717.68847 (49.0%), 1718.69183 (15.9%), 1717.68601 (6.2%), 1718.68936 (6.1%), 1719.69518 (3.8%), 1719.69272 (3.0%), 1716.68804 (1.2%), 1717.69139 (1.2%), 1716.68598 (1.1%), 1717.68933 (1.1%), 1720.69607 (1.0%)

SMILES:
CC(COC(C(COC(C1(COC(C2=CC=CC=C2)OC1)C)=O)(COC(C3(COC(C4=CC=CC=C4)OC3)C)=O)C)=O)(COC(C(CO C(C5(COC(C6=CC=CC=C6)OC5)C)=O)(COC(C7(COC(C8=CC=CC=C8)OC7)C)=O)C)=O)CO/C(C(COC(C9(COC(C %10=CC=CC=C%10)OC9)C)=O)(COC(C%11(COC(C%12=CC=CC=C%12)OC%11)C)=O)C)=[O+]/[Na]

m/z:* 1715.681763

*Note: There is a small discrepancy in the calculated mass of S1325 and S1716 from chemdraw to that listed here and in Table 1. This value (Table 1) is more accurate.
